# Cross-cultural validation of acculturation measures: Expanding the East Asian acculturation framework for global applicability

**DOI:** 10.1371/journal.pone.0310351

**Published:** 2025-03-11

**Authors:** Erhabor Sunday Idemudia, Constance Karing, Lawrence Ejike Ugwu

**Affiliations:** 1 Faculty of Humanities, North-West University, Potchefstroom, South Africa; 2 Institute fur Psychologie, Friedrich-Schiller University, Jena, Germany; The Open University of Israel, ISRAEL

## Abstract

In a globalised world, understanding acculturation, the process by which individuals adapt to new cultural environments, is crucial, especially in multicultural societies experiencing increased migration. The East Asian Acculturation Measure (EAAM), based on Berry’s acculturation model, has been a cornerstone for assessing acculturation strategies among East Asian populations in the United States; however, its cultural specificity limits utility in broader contexts. This study addresses this gap by adapting and validating the EAAM for diverse populations, producing the Shortened Adapted Acculturation Scale (SAAS). Across two phases involving 490 university students from 87 nationalities in Germany and 329 university students from 25 nationalities in South Africa, both Confirmatory Factor Analysis (CFA) and Exploratory Factor Analysis (EFA) identified a five-factor structure: Social Disconnection, cultural adaptation, Social Perception, Interpersonal Comfort, and Language Integration. The SAAS showed high internal consistency and measurement invariance across genders. These results highlight the importance of culturally adapting psychological measures to ensure their relevance and reliability in global contexts. The SAAS offers practical benefits for clinicians, educators, and policymakers who serve multicultural populations. By illuminating specific dimensions of acculturation, the scale can help identify areas where targeted interventions such as mental health counselling, cultural orientation programs, or inclusive campus policies may foster better social integration and well-being. Although the present study focused on structural validity, future research should examine the SAAS’s predictive utility for mental health and social integration outcomes. These findings contribute to cross-cultural psychology and underline the need to refine and validate tools for assessing acculturation in an increasingly interconnected world.

## Introduction

In today’s globalised world, understanding acculturation—the process by which individuals adapt to new cultural environments—is essential. Acculturation research often builds on Berry’s [1,2] bidimensional framework, categorising acculturation strategies into four types: assimilation, separation, integration, and marginalisation. Berry’s model is not merely a collection of survey items; it is a theoretical framework that describes how individuals balance their identification with their culture of origin and the host culture. To capture this balance, Berry’s model requires administering parallel scales for each cultural domain, where individuals respond separately to questions about their origin and host cultures. Acculturation strategies are then derived by comparing responses across these two domains, categorising respondents based on high or low attachment to each culture. For example, high attachment to both cultures signifies integration, whereas high attachment only to the host culture indicates assimilation.

The East Asian Acculturation Measure (EAAM), developed by Barry [3], is an empirical adaptation inspired by Berry’s framework, specifically designed to capture acculturation patterns among East Asian immigrants in the United States. While Berry’s model theoretically requires a bidimensional approach with parallel measures for each cultural domain, Barry’s EAAM uses a unidimensional structure focusing only on the host-cultural context (i.e., the United States). Thus, the EAAM operationalises Berry’s four acculturation strategies(assimilation, separation, integration, and marginalisation) within a single cultural frame, as experienced by East Asian populations in the United States. Despite the value of Barry’s EAAM in understanding acculturation within this specific demographic, its design reflects host-culture-specific dynamics, limiting its applicability to other cultural groups and contexts, such as populations in South Asia, Africa, or Latin America, and making it less suited for cross-cultural comparisons in its current form.

This study aims to adapt and validate the EAAM for broader cultural applicability across diverse populations, developing the Shortened Adapted Acculturation Scale (SAAS). By modifying specific items for cultural inclusivity, this research addresses contemporary globalisation trends and the increasing diversity of migrant populations, filling a gap in acculturation research by providing a measure that can be used across various cultural contexts. The SAAS offers researchers and practitioners a culturally sensitive tool that facilitates a deeper understanding of how diverse populations navigate acculturation processes in non-U.S. settings, such as Germany and South Africa. This adaptation is timely and relevant given the growing need for validated acculturation measures that extend beyond a single cultural group and can support initiatives in clinical, educational, and policy settings aimed at culturally diverse populations.

### Methods

This study comprised two phases aimed at adapting and validating the East Asian Acculturation Measure (EAAM) for use across diverse cultural contexts, ultimately resulting in the Shortened Adapted Acculturation Scale (SAAS). Each phase addressed specific goals: Study 1 explored the EAAM’s factor structure and identified a culturally relevant adaptation, while Study 2 validated the SAAS and tested measurement invariance across genders.

## Study 1: Testing the psychometric properties of the EAAM in Germany

Study 1 aimed to assess the EAAM’s factor structure and cultural applicability among a diverse sample of international students in Germany. Given the EAAM’s East Asian-centric design, the study explored modifications necessary for adapting the scale to broader cultural contexts.

### Participants

The participants were 490 university students from 87 nationalities enrolled in German universities. The sample’s age range was 18–54 years (M =  26.07, SD =  4.38), with 60.7% identifying as male. Eligibility required non-German nationality and a minimum of six months’ residence in Germany, ensuring sufficient exposure to the host culture. Recruitment involved university email lists, social media, and on-campus posters. Given the sample’s cultural heterogeneity, we refrained from making cross-national comparisons.

### Measures

#### Instrument Section for the East Asian Acculturation Measure (EAAM).

The East Asian Acculturation Measure (EAAM) was initially developed for East Asian populations in the U.S., and based on Berry’s acculturation model, the EAAM includes 29 items across four acculturation dimensions: Assimilation, Separation, Integration, and Marginalisation. Items are rated on a 7-point Likert scale. Notably, Berry’s categories describe respondent acculturation strategies, independent of any specific measurement instrument or item groups, necessitating careful adaptation of the EAAM for diverse samples. Examples of items modified: “I get along better with [*country of residence*] than people from my country,” and “Most of my friends at work/school are [*country of residence*].”

### Procedure

The study adopted a cross-sectional design targeting international students at German universities to assess acculturative strategies. Eligible participants included international students currently enrolled in German universities, aged 18 or older, who had lived in Germany for at least six months. Recruitment was conducted via university email lists, social media, and campus posters. Participants accessed the survey through a QR code or direct link. The survey, hosted on SoSci Survey and expected to take 15 to 20 minutes, collected data on demographics and acculturative strategies.

Informed consent was obtained electronically, and participants were assured confidentiality and their right to withdraw. The Friedrich Schiller University Jena Ethics Committee (FSV 23/049) granted ethical approval, and the study adhered to strict ethical guidelines. Data were anonymised, and the study was classified as low-risk. Measures were implemented to address any potential psychological discomfort, including providing counselling resources if needed.

### Data analysis

The initial analysis utilised Confirmatory Factor Analysis (CFA) to test the EAAM’s original four-factor model, evaluating fit indices including Chi-square (χ²), Root Mean Square Error of Approximation (RMSEA), and Comparative Fit Index (CFI). Due to suboptimal fit, Exploratory Factor Analysis (EFA) was conducted, allowing for data-driven identification of a factor structure more suited to this culturally diverse sample. EFA was performed using principal axis factoring with oblique rotation to capture potential correlations among factors. This analysis led to a refined five-factor structure, which informed the Shortened Adapted Acculturation Scale (SAAS) development. No new methods of factor extraction were introduced.

### Result

A Confirmatory Factor Analysis (CFA) was conducted to test the four factors of the EAAM. The 4-factor model produced a chi-square value of χ²(224) =  1371.74, p < .001. The fit indices were: Standardized Root Mean Square Residual (SRMR) = .06, Goodness of Fit Index (GFI) = .85, Normed Fit Index (NFI) = .87, Non-Normed Fit Index (NNFI) = .88, Comparative Fit Index (CFI) = .89, and Root Mean Square Error of Approximation (RMSEA) = .082. All the fit indices like SRMR, NFI, and CFI were below the acceptable ranges of.90 and above, and the RMSEA exceeds the commonly accepted threshold of 0.08, indicating that the model does not adequately fit the data (see Table 1). Therefore, an exploratory factor analysis was conducted to identify possible factors that fit the data.

**Table 1:** The confirmatory factor analysis of the EAAM.

Model description	chi-square	DF	SRMR	GFI	NFI	NNFI	CFI	RMSEA
4-FACTOR	1371.74	224	.06	.85	.87	.88	.89	.082

Root Mean Squared Error of Approximation (RMSEA), the root mean square residual (RMR), the standardised root mean squared residual (SRMR), the Comparative Fit Index (CFI), and the Goodness of Fit Index (GFI), Normed Fit Index (NFI), Non-Normed Fit Index (NNFI)

Principal Axis Factoring (PAF) using the original 29 items. The result of the PAF revealed five factors, with 18 items loading on five factors. The other eleven items had lower loadings or cross-loaded on two or more factors and were removed. This process helped in reducing item redundancy. The criteria for loading is a minimum of 0.3 on only one factor. The PAF generated five factors explaining 51.88% of the variance for the complete set of variables. An analysis of the Kaiser–Meyer Olkin (KMO) test of sampling adequacy suggested that the calibration sample was suitable for PAF (KMO =  0.86) with the Bartlett’s Test of Sphericity significant (χ2 =  8600.87; df =  406; p <  0.001). Four items loaded on factor one explained 20.84% of the total variance, four items loaded on factor two explained 14.78% of the variance, factor three had four items too, which explained 7.12% variance, the fourth factor with three items explained 5.87%, and fifth factor with three items explained 3.96% of the total variance.

**Fig 1:**
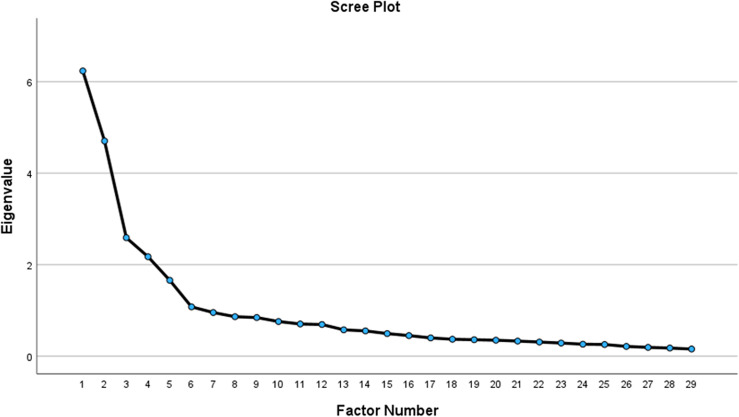
Scree plot for the Five-factor SAAS.

The scree plot further reveals the analysis components’ breaking point. The eigenvalue is plotted against the number of components included in the analysis. Fig 1, shows that the eigenvalue was above 1.00 only among the first five factors.

## Study 2: Validation and measurement invariance of the SAAS in South Africa

The goal of Study 2 was to validate the SAAS identified in Study 1 and to test measurement invariance across genders in a South African sample. Ensuring consistent measurement across genders supports the SAAS’s utility for diverse populations.

### Participants

Study 2 involved 329 university students from 25 nationalities studying in South Africa, aged between 16 and 54 (M =  24.04, SD =  6.08), with an almost equal gender distribution (50.5% female, 49.5% male). Similar to Study 1, inclusion criteria required students to be non-South African nationals with a minimum of six months’ residence in South Africa. Participants were recruited through university communications and social media. This sample reflects diverse cultural experiences within the South African context, precluding claims about cross-national comparability.

### Measures

#### Instrument Section for the Shortened Adapted Acculturative Strategy (SAAS).

The SAAS is an 18-item self-report measure adapted from the EAAM by Barry [3] that measures the acculturative strategy of participants. The SAAS is a multidimensional aspect of acculturation [Social Disconnection/social alienation/marginalisation (4-items), Cultural adaptation/social integration (4-items), Cross-cultural social dynamic/cultural affiliation/social perception (4-items), Relationship Preference/interpersonal comfort (3-items) and Cultural expression & Language proficiency/cultural identity & Language dynamic (3-items)] among a general population. The items are rated on a 7-point Likert-type scale ranging from 1 (strongly disagree) to 7 (strongly agree). Examples of items modified: “I get along better with [*country of residence*] than people from my country,” and “Most of my friends at work/school are [*country of residence*].” The total score for each subscale is calculated by summing the relevant item scores.

#### Acculturative Stress Scale for International Students (ASSIS).

The Acculturative Stress Scale for International Students (ASSIS) is a 36-item self-report scale developed by Sandhu and Asrabadi [4]. It was utilised to measure the acculturative stress experienced by international students. It was explicitly designed to assess the psychological challenges that international students face as they adjust to a new cultural environment. The ASSIS addresses a broad range of stressors that contribute to acculturative stress, making it a comprehensive tool for understanding the unique experiences of this population. The ASSIS comprised six primary factors representing different dimensions of acculturative stress [Perceived Discrimination (8 items), Homesickness (4 items), Perceived Hate (5 items), Fear (4 items), Stress Due to Change/Culture Shock (3 items), Guilt (2 items). Each item is rated on a 5-point Likert-type scale ranging from 1 (strongly disagree) to 5 (strongly agree), with higher scores indicating greater levels of acculturative stress.

### Procedure

The second study also adopted a cross-sectional design. It focused on international students at South African universities to assess SAAS and acculturative stress. All participants were international students enrolled in South African universities, aged 18 or older, and residents of South Africa for at least six months. Participants were purposively sampled.

Recruitment was conducted through university email lists, social media, and campus posters, with the survey accessible via a QR code or direct link. The survey, conducted on SoSci Survey and expected to take 20 to 25 minutes, gathered data on demographics, acculturative strategies, and stress. Informed consent was obtained electronically, ensuring participants’ confidentiality and their right to withdraw. The study received ethical approval from the North-West University BaSSREC (NWU-01085-22-S7-01) and adhered to strict ethical guidelines. Data were anonymised, and the study was classified as low-risk. Provisions were in place to address any psychological discomfort, including access to counselling support.

### Data analysis

CFA tested the five-factor model derived from Study 1, assessing the SAAS’s validity in this new sample. Measurement invariance across genders was tested using multiple-group CFA to evaluate configural, metric, and scalar invariance, ensuring that the SAAS consistently measures acculturation for both male and female respondents. Fit indices such as Chi-square (χ²), RMSEA, and CFI were used to assess model fit data [5,6]. Measurement invariance was essential to confirm the SAAS’s applicability across demographic subgroups. The model fit of the CFA was estimated using AMOS Graphics (version 29).

#### Multiple-group CFA of invariance across gender.

Measurement invariance examines whether the assessment of latent constructs is consistent across different groups, as discussed by Cheung and Rensvold [7] and Kline [8]. This process involves evaluating three critical levels of invariance: configural, metric, and scalar. Configural invariance tests whether the same factor structure is applicable across groups, indicating that the observed variables represent the same pattern of latent constructs in different populations. If configural invariance is supported, it suggests that the basic model holds across groups. However, it does not imply that the relationships between latent constructs and observed variables are identical across those groups [9, 10].

Metric invariance is examined next to ensure the constructs have the same meaning across groups. This level of invariance assesses whether the factor loadings are equivalent, meaning that the constructs are interpreted similarly across different populations. Without metric invariance, comparing latent means across groups is invalid because the constructs may be understood differently [8,10].

Finally, scalar invariance is tested to determine whether the intercepts of the observed variables are consistent across groups. This is necessary for comparing latent means, as it ensures that the groups have the same baseline level for the latent constructs. If scalar invariance holds, it suggests that differences in means reflect true differences in the latent constructs rather than differences in measurement [10]. The results of these tests for measurement invariance are presented in Table 4.

**Table 2:** Confirmatory factor analysis of the Five-subscales of SAAS.

Model description	chi-square	DF	SRMR	GFI	NFI	NNFI	CFI	RMSEA
5-FACTOR	638.95	260	.17	.86	.90	.91	.92	.061

Root Mean Squared Error of Approximation (RMSEA), the root mean square residual (RMR), the standardised root mean squared residual (SRMR), the Comparative Fit Index (CFI), and the Goodness of Fit Index (GFI), Normed Fit Index (NFI), Non-Normed Fit Index (NNFI)

**Table 3:** Confirmatory Structure of the Five-Dimensional Model.

Factors	Items	Factor loadings	α	CR	AVE
F1	26	0.838	0.90	0.91	0.71
	27	0.789			
	28	0.931			
	29	0.797			
F2	04	0.831	0.92	0.92	0.75
	05	0.901			
	06	0.877			
	07	0.859			
F3	10	0.740	0.88	0.88	0.64
	11	0.782			
	13	0.848			
	14	0.837			
F4	18	0.814	0.89	0.89	0.73
	19	0.926			
	20	0.819			
F5	01	0.839	0.87	0.87	0.68
	02	0.768			
	03	0.868			

F1-Social Disconnection; F2-Cultural adaptation; F3-Social perception; F4- Interpersonal comfort; F5- Language integration; α- Cronbach’s alpha; CR- Composite Reliability; AVE-Average Variance Extracted.

**Table 4:** Discriminant validity of the SAAS.

HTMT	F1	F2	F3	F4	F5
**F1**					
**F2**	0.241				
**F3**	0.376	0.250			
**F4**	0.234	0.285	0.351		
**F5**	0.173	0.065	0.372	0.342	

HTMT- Hetrotrait-Monotrait Ratio; F1-Social Disconnection; F2-Cultural adaptation; F3-Social perception; F4- Interpersonal comfort; F5- Language integration

### Result

#### Confirmatory factor analysis of the SAAS five-factor model.

**Fig 2:**
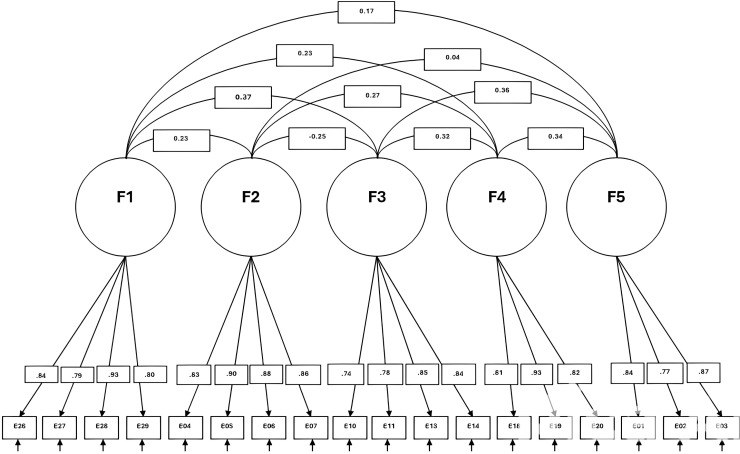
Five-dimensional model for the five subscales of the SAAS.

A five-dimensional model of the scale was tested. Each item was constrained to load on the hypothesised dimension. The factor analysis of the data yielded goodness-of-fit indices that support the five-dimensional structure of the adapted scale. The Chi-square goodness-of-fit was significant, with χ2 =  638.95; df =  260; p =  0.001. Furthermore, the other fit indices were all within acceptable limits, with GFI =  0.90, CFI =  0.92, NNFI =  0.91, and RMSEA =  0.061. Recent guidelines suggest the use of the Non-Normed Fit Index (NNFI or TLI), Comparative Fit Index (CFI), and Root Mean Square Error of Approximation (RMSEA) for evaluating model fit. Current standards recommend that NNFI and CFI values above 0.95 indicate a good model fit, and RMSEA values below 0.06 are considered acceptable [5,8,13]. Thus, we considered our model as acceptable. We examined the internal consistency indices of the dimensions (see Fig 2. and Table 2).

A measurement model evaluating the scale’s psychometric properties revealed a five-factor structure (see Table 3). The analysis assessed each factor’s internal consistency, reliability, and validity.

The first factor, social disconnection, comprised four items demonstrating strong loadings, indicating that the factor reliably captured the underlying construct. Similarly, Cultural adaptation included four items, all of which showed excellent reliability, confirming the robustness of this factor in representing the adaptation process.

Social perception was also identified as a factor consisting of four items, with results showing good reliability and construct validity. This factor effectively measures individuals’ perceptions within social contexts.

Interpersonal comfort emerged as a factor comprising three items, exhibiting strong internal consistency and validating it as a reliable measure of comfort in interpersonal interactions.

Lastly, language integration comprises three items, each demonstrating high reliability and confirming the factor’s ability to represent language skill integration accurately.

The results indicate that all five factors demonstrate strong internal consistency and reliability. These findings suggest that the factors are valid representations of their respective constructs, capturing a substantial portion of the variance in the items. This comprehensive evaluation reaffirms the scale’s reliability and validity, providing security for measuring these specific psychological constructs.

Discriminant validity was assessed using the Heterotrait-Monotrait ratio (HTMT) to ensure that each construct within the scale is distinct from the others (see Table 4). The analysis revealed that all HTMT values were well below the conservative threshold of 0.85, typically recommended for establishing discriminant validity.

The results confirm that the constructs measured by the scale are sufficiently distinct, demonstrating strong discriminant validity. This suggests that the scale effectively captures unique dimensions of the constructs under investigation, ensuring that each factor is not merely a reflection of the others but represents a separate and meaningful concept within the broader framework of the study.

A bivariate correlation analysis assessed the relationship between the subscales of the newly validated scale and acculturative stress, focusing on the expected negative correlations (see Table 5). The Cultural Adaptation subscale showed significant negative correlations with acculturative stress factors, supporting the expected inverse relationship and indicating convergent validity. However, other subscales, such as Social Disconnection and Language Integration, showed weak positive correlations, contrary to the hypothesised negative relationship. These results suggest that while Cultural Adaptation aligns with theoretical expectations, the other subscales do not consistently demonstrate the anticipated negative relationship with acculturative stress, indicating that further investigation may be needed.

**Table 5:** Convergent between the subscales of SAAS and acculturative stress.

	M	SD	1	2	3	4	5	6	7	8	9	10	11	12
Perceived discrimination (1)	23.53	8.08	--											
Homesickness (2)	12.76	3.973	.72**	--										
Perceived hate (3)	14.58	5.85	.94**	.72**	--									
Fear (4)	11.76	3.94	.88**	.69**	.81**	--								
Stress (5)	8.98	3.21	.79**	.80**	.80**	.71**	--							
Guilt (6)	5.74	2.40	.76**	.73**	.76**	.68**	.75**	--						
Miscellaneous (7)	29.39	10.34	.93**	.77**	.91**	.84**	.80**	.78**	--					
Social disconnect (8)	14.18	5.92	.25**	.11 *	.25**	.24**	.14**	.17**	.29**	--				
cultural adaptation (9)	12.89	5.97	-.22**	-.29**	-.23**	-.21**	-.27**	-.22**	-.21**	.19**	--			
Social perception (10)	17.73	6.14	.23**	.26**	.22**	.26**	.26**	.23**	.24**	.33**	-.23**	--		
Interpersonal comfort (11)	14.27	4.44	-.13 *	-.03	-.18**	-.09	-.12 *	-.15**	-.12 *	.20**	.23**	.34**	--	
Language integration (12)	14.93	4.78	.16**	.07	.13 *	.13 *	.12 *	.05	.11 *	.17**	.04	.35**	.31**	--

*  p < .05, **p < .001.

### Measurement invariance

A series of nested models were tested to evaluate the scale’s measurement invariance across genders (see Table 6). The Configural invariance (Model 1), serving as the baseline model, yielded a chi-square value of χ²(260) =  638.95, Comparative Fit Index (CFI) =  0.92, Gamma Hat =  0.91, and McDonald’s Non-Centrality Index (Mc NCI) =  0.63. These results indicate a good fit, suggesting that the same factor structure is valid across both male and female groups.

**Table 6:** Results of tests for measurement invariance across genders.

Model	Model description	chi-square	df	CFI	gamma hat	Mc NCI	Δchi-square	Δdf	sig.	ΔCFI	ΔMc NCI	Δgamma hat
1	Configural invariance	638.95	260	0.92	0.91	0.63	–	–	–	–	–	–
2	Metric invariance	661.29	273	0.92	0.90	0.62	22.34	13	0.06	0.002	0.01	0.01
3	Scalar invariance	669.93	283	0.92	0.90	0.62	8.64	10	0.57	0.00	0.001	0.002

Mc NCI, McDonald’s Non-Centrality Index; CFI, Comparative Fit Index.

The finding of scalar invariance indicates that observed differences in SAAS scores between men and women can be interpreted as genuine differences in the underlying constructs, rather than artifacts of measurement bias. This practical significance enables meaningful comparisons of average subscale scores across genders, supporting equitable research and intervention design by confirming that the SAAS functions similarly for both male and female respondents.

Metric invariance (Model 2) was tested by constraining the factor loadings to be equal across genders. The fit indices for this model were χ²(273) =  661.29, CFI =  0.92, Gamma Hat =  0.90, and Mc NCI =  0.62. The chi-square difference test comparing the configural and metric models yielded Δχ²(13) =  22.34, p =  0.06. Although the chi-square difference approached significance, the changes in CFI (ΔCFI =  0.002), Gamma Hat (ΔGamma Hat =  0.01), and Mc NCI (ΔMc NCI =  0.01) were minimal, supporting metric invariance. This indicates that the factor loadings are equivalent across genders, meaning the construct has the same meaning for both men and women.

Scalar invariance (Model 3) was assessed by further constraining the item intercepts to be equal across genders. This model produced a chi-square value of χ²(283) =  669.93, CFI =  0.92, Gamma Hat =  0.90, and Mc NCI =  0.62. The chi-square difference between the metric and scalar models was Δχ²(10) =  8.64, p =  0.57, which was not statistically significant. Additionally, changes in CFI (ΔCFI =  0.00), Gamma Hat (ΔGamma Hat =  0.002), and Mc NCI (ΔMc NCI =  0.001) were negligible, indicating that scalar invariance is supported. This suggests that the item intercepts are consistent across genders, allowing for comparing latent means between men and women.

The results indicate that the scale demonstrates configural, metric, and scalar invariance across gender. This implies that the factor structure, factor loadings, and item intercepts are consistent for both male and female groups, making meaningful comparisons of latent means between genders. Although the chi-square difference for the metric invariance test was close to significance (p =  0.06), the minimal changes in other fit indices (CFI, Gamma Hat, and Mc NCI) suggest that metric invariance holds. The support for scalar invariance confirms that differences in latent means between genders can be interpreted as true differences rather than measurement artefacts.

An independent samples t-test was further conducted to explore gender differences across five psychological factors: Social Disconnection, Cultural Adaptation, Social Perception, Interpersonal Comfort, and Language Integration. The analysis revealed no significant differences between males and females on any of the factors. Both genders exhibited similar scores for each factor, indicating that gender does not significantly influence these constructs in this sample. This suggests that the psychological traits measured by these factors are consistent across genders.

## Discussion

This study adapted and validated the East Asian Acculturation Measure (EAAM) to create the Shortened Adapted Acculturation Scale (SAAS), suitable for diverse cultural contexts outside the original East Asian population. Through rigorous psychometric evaluations, the SAAS emerged as a robust tool comprising five distinct dimensions: Social Disconnection, Cultural Adaptation, Social Perception, Interpersonal Comfort, and Language Integration. This multidimensional structure better captures the complexities of acculturation in varied cultural settings, underlining the need for culturally adaptable tools that address unique acculturation experiences.

The initial CFA results for the original EAAM structure highlighted limitations in applying a culturally specific tool to a heterogeneous sample, consistent with previous research emphasising the necessity of cultural adaptation in psychological assessment tools [11]. The EFA results indicated that a five-factor structure provided a more appropriate fit, reflecting the unique acculturative experiences of a global sample and revealing additional dimensions, such as Social Disconnection and Language Integration, which were not initially captured in the EAAM. This enhanced model underlines the complexity of cultural adaptation processes, affirming that acculturation involves a broader range of experiences than the original dimensions of Assimilation, Integration, Separation, and Marginalisation could capture.

### Cultural adaptation and practical implications

The development of the SAAS required not only a psychometric re-envisioning of the original EAAM but also a careful cultural adaptation of its items [12]. Whereas the EAAM’s language and examples were tailored to East Asian populations in the U.S., the SAAS reframes and broadens these references to accommodate a wider range of contexts. For example, items originally referencing “Americans” or “U.S.-born friends” were modified to more neutral terms such as “people from [country of residence],” allowing individuals of varied nationalities and cultural backgrounds to relate the scale’s content to their lived experiences. Similarly, items that highlighted culture-specific norms or values were updated or replaced with language emphasizing more universal domains of social interaction, perceived cultural differences, and language use. These changes ensured that the SAAS better captures the realities faced by diverse populations, including those studying or working in countries like Germany or South Africa, without presupposing familiarity with a specific cultural setting.

By embracing a more inclusive and context-sensitive approach to item wording, the SAAS holds practical value for clinicians, educators, and policymakers working with increasingly multicultural and mobile populations. For instance, mental health professionals can use the SAAS to identify areas of social disconnection or language-related challenges that may contribute to acculturative stress, subsequently guiding more tailored support services. Educational institutions could employ the SAAS to design culturally responsive programs that facilitate smoother integration for international students, thereby improving their academic engagement and overall well-being. Similarly, policymakers and community organizations can draw on SAAS data to inform initiatives fostering intercultural dialogue, social cohesion, and community-based resources that support culturally diverse groups. In this way, the SAAS not only advances theoretical understanding of acculturation processes beyond East Asian contexts but also serves as an applied tool for promoting successful adaptation in an increasingly globalized world.

### Predictive validity and future research directions

While the SAAS demonstrated robust structural validity, measurement invariance across genders, and internal consistency, the present study focused primarily on its factor structure rather than its ability to predict meaningful outcomes. As evidenced by the inconsistent correlations between the SAAS subscales and measures of acculturative stress where Cultural Adaptation aligned with theoretical expectations, but Social Disconnection did not, further exploration is needed to establish the scale’s predictive utility. Some individuals may not find social disconnection inherently stressful, especially if they have alternative coping strategies, online communities, or resilient cultural enclaves. In some cases, social withdrawal may even serve as a temporary adaptive response, reducing immediate stress. Methodological and cultural factors may also play a role. Cross-sectional data can capture participants at different stages of adjustment, and cultural differences in how social ties are understood may influence the results.

Future research should examine how SAAS scores relate to long-term psychological and social outcomes, such as mental health indicators (e.g., depression, anxiety, or well-being), successful social integration, academic performance, or professional adaptability.

To achieve this, researchers could employ longitudinal or prospective study designs that follow participants over time as they navigate new cultural environments. Such approaches would allow investigators to link baseline SAAS scores to changes in psychological well-being, social network formation, language acquisition, and community involvement. Incorporating well-validated external criteria (e.g., clinical assessments of mental health, objective indicators of cultural adaptation, or measures of social support) will provide stronger evidence of the SAAS’s predictive validity. Moreover, examining the scale’s performance across a range of demographic groups, cultural contexts, and life stages will clarify its generalizability and practical relevance.

By testing the SAAS’s ability to predict key outcomes, future research can move beyond merely describing acculturation processes to identifying factors that contribute to successful adaptation or heightened vulnerability. Establishing predictive validity would significantly enhance the SAAS’s utility in clinical, educational, and policy settings, offering practitioners a valuable tool for early intervention and support programs aimed at improving mental health and social integration among culturally diverse populations [14].

### Limitations of the study

This study is subject to several limitations. First, although the participant pool was diverse in terms of nationality and cultural background, it primarily consisted of university students from Germany and South Africa. University students may not accurately reflect broader acculturation experiences due to their relatively young age, higher educational attainment, and unique social and institutional contexts. As such, the current findings may not generalize well to other demographic groups such as working professionals, older adults, individuals with varying educational levels or socioeconomic statuses, or those residing in non-academic settings. Future research should validate the SAAS among more heterogeneous samples, including migrants in different stages of life, refugees, and those from rural or non-institutional communities. Such efforts would help establish the scale’s applicability and robustness across a wider range of cultural contexts, ultimately strengthening the SAAS as a comprehensive tool for assessing acculturation processes in diverse global populations.

Second, although gender-based measurement invariance was established, other demographic factors such as ethnicity, migration history, and socio-economic status were not tested. These variables may influence acculturation experiences and should be considered in future validation studies to improve the SAAS’s applicability across broader social contexts.

Lastly, while reducing items from the original EAAM improved the SAAS’s internal consistency, it potentially limits the measure’s comprehensiveness. The decision to reduce items was made to ensure the SAAS remained a manageable and reliable tool, yet further validation should explore whether the retained items comprehensively capture acculturation constructs across cultures. This would further strengthen confidence in the SAAS’s use as a standardised tool for measuring acculturation beyond East Asian populations.

## Conclusion

The adaptation and validation of the SAAS mark a significant contribution to cross-cultural psychology by providing a psychometrically sound, culturally adaptable measure of acculturation. The SAAS offers researchers and practitioners a refined tool for assessing acculturation among diverse populations, enabling insights into individuals’ specific acculturative challenges in multicultural settings. As globalisation increases cultural diversity worldwide, tools like the SAAS will become essential for understanding and supporting individuals navigating complex cultural landscapes. Future research should continue examining the SAAS’s predictive validity and applicability in broader populations, enhancing its role as a key instrument in acculturation research.

## Supporting Information

S1 Data Dataset for SA International students (CSV). (CSV)

S2 Data Dataset for Germany international students (CSV). (CSV)

S3 Data Inclusivity in global research questionnaire. (DOCX)
